# Impurity Segregation and Nanoparticle Reorganization of Indium Doped MgO Cubes

**DOI:** 10.1002/cnma.201900077

**Published:** 2019-04-03

**Authors:** Matthias Niedermaier, Chatpawee Taniteerawong, Thomas Schwab, Gregor Zickler, Johannes Bernardi, Oliver Diwald

**Affiliations:** ^1^ Department of Chemistry and Physics of Materials University of Salzburg Jakob-Haringer-Strasse 2a 5020 Salzburg Austria; ^2^ University Service Centre for Transmission Electron Microscopy Technische Universität Wien 1040 Vienna Austria

**Keywords:** impurity admixture, indium exsolution, grain boundary engineering, transparent conductive ceramics, phase separation

## Abstract

Metal oxide nanocomposites are non‐equilibrium solids and promising precursors for functional materials. Annealing of such materials can provide control over impurity segregation and, depending on the level of consolidation, represents a versatile approach to engineer free surfaces, particle‐particle interfaces and grain boundaries. Starting with indium‐magnesium‐oxide nanoparticle powders obtained via injection of an indium organic precursor into the magnesium combustion flame and subsequent particle quenching in argon, we investigated the stability of the trivalent In^3+^ ions in the host lattice of MgO nanoparticles by determining grain growth, morphology evolution and impurity segregation. The latter process is initiated by vacuum annealing at 873 K and can be tracked at 1173 K on a time scale of minutes. In the first instance the surface segregated indium wets the nanoparticle interfaces. After prolonged annealing indium evaporates and leaves the powder via the gas phase. Resulting MgO nanocubes are devoid of residual indium, regain their high morphological definition and show spectroscopic fingerprints (UV Diffuse Reflectance and Photoluminescence emission) that are characteristic of electronically unperturbed MgO cube corner and edge features. The results of this combined XRD, TEM, and spectroscopy study reveal the parameter window within which control over indium segregation is used to introduce a semiconducting metal oxide component into the intergranular region between insulating MgO nanograins.

## Introduction

Impurity segregation in solids generates strong gradients in the chemical composition between surfaces, interfaces and grain boundaries, on the one hand, and the bulk of the grains, on the other. In the field of ceramics there is increasing awareness that the adjustment and controlled manipulation of interface composition have promising effects on reactivity,^1^ grain boundary resistivity,^2^ thermoelectric properties,^3^ enhanced stability against low temperature degradation^4^ and many properties more, that determine the materials’ performance of functional and structural nanoceramics.^5^ Stability of admixed cations and associated segregation behavior have been extensively studied on MgO crystals and polycrystals.^6^ This is mostly due to the host's simple structure and its pronounced ionicity. The surface energetics of the alkaline earth oxides are very different from those of other oxides like alumina, titania and iron oxide, since for MgO the {100} planes are very much lower in energy than the other low index surfaces such as {110} and {111}.^7^ Impurity admixture and annealing induced surface segregation can affect this hierarchy of surface energies and may stabilize particle morphologies with higher concentrations of high index microfacets or step edges and other than cubes. Hence, the investigation of impurity segregation in a well‐studied metal oxide such as MgO may reveal interesting new research strategies when applied to nanocrystals. Processes like i) coarsening, defect chemistry and phase separation at a single particle level^8^ or ii) particle morphology transformations by impurity induced surface energy changes can be addressed. For this purpose, we used individual MgO nanoparticles as “particulate test vessels” for the study of defect chemistry and solid state reactions in confined space.^8^

Our interest in the binary In_2_O_3_−MgO system is reasoned by earlier observations by others:^9^ on single crystalline MgO substrates indium oxide films, which are produced by metal‐organic chemical vapor deposition, were found to grow in epitaxial relationships. Being relevant for transparent conductive oxide (TCO) related applications, MgIn_2_O_4_ 10 has shown good electrode performance in electronic devices. Elevated temperatures and/or electric fields, which typically are part of the working conditions of electroceramic devices, favor solid‐state reactions. Moreover, the MgO−In_2_O_3_ interface can undergo a spinel formation reaction

(1) $$MgO+{In}_{2}O_{3}\;\overset{\Delta T\;}{\underset{}{\to }}\;Mg{In}_{2}O_{4}$$

where the associated kinetics and morphological aspects were investigated by Korte et al.^11^ Corresponding findings underline the determining role of grain boundaries as fast diffusion paths sustaining the solid‐state reaction. In order to fully realize the range of device applications where metal oxide nanocomposites serve as precursor structures, it is necessary to understand compositional, structural and topographical changes inside the intergranular regions that occur during nanoparticle processing spanning the range from particle synthesis to sintering.^12^ New material concepts and insights would result from the rationalization of respective transformation steps and are highly influential to the resulting materials performance.

This work represents a first study on the nanocrystalline and particle based In−Mg−O system and focuses on the stability of the In^3+^ ions within the MgO host lattice. Comparison of structure and composition of the composite nanoparticles, either as‐synthesized or after thermal processing, reveal trends, that are key for the later exploitation of their functional properties.^13^ The choice of two processing temperatures, T=873 K and 1173 K, is based on previous observations:^8^ oxygen treatment at 873 K was found to be effective for complete carbon contaminant elimination, whereas vacuum annealing at 1173 K leads to dehydroxylated MgO nanoparticle surfaces.^14^ The investigation of annealing‐induced particle coarsening, on the one hand, and assessment of particle morphologies, on the other, provide important insights into the stability of the host nanocrystals containing In^3+^ ions as aliovalent impurities and with ionic radius that is larger ($r^{In\left(III\right)}$ =80 pm) than that of Mg^2+^ ($r^{Mg\left(II\right)}$ =72 pm) inside the cubic host lattice.^15^

## Results and Discussion

**Figure 1:**
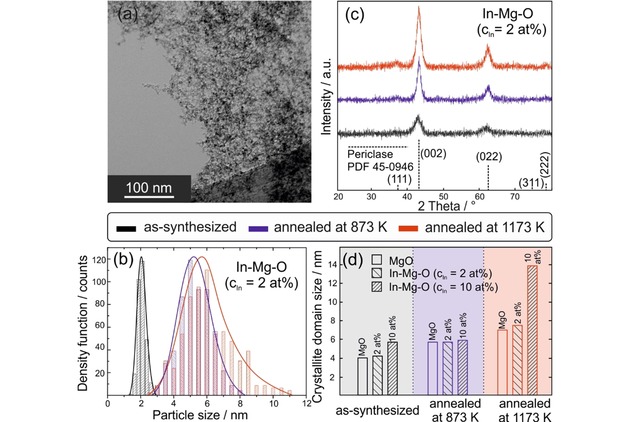
Low magnification TEM image (a) of a In−Mg−O sample (c_In_=2.0 at%) showing the high dispersion of the nanocrystal powder as observed for as‐synthesized samples of variable concentration. Particle size distributions (b) and XRD powder patterns (c) acquired on In−Mg−O nanoparticle powders either as‐synthesized (black) or after thermal processsing to T=873 K (blue) or 1173 K (red)) in alternating atmospheres (vacuum and oxygen). Crystalline domain sizes (d) were determined from the (002) related reflection widths using the Scherrer equation.

We start with as‐synthesized powders of In−Mg−O nanocomposite particles that were grown using a newly developed chemical vapor synthesis approach (Figure S1, Supporting Information).^8^,^16^ The process employs the decomposition of an indium organic precursor inside the Mg vapor combustion flame which is associated with a combustion reaction that exhibits sufficiently high exothermicity and high temperatures (≈2600 K) to completely convert the precursor components into metal oxides. Using this approach, we obtain powders of highly dispersed and crystalline nanoparticles (Figure 1a) with an extremely homogeneous distribution of size (Figure 1b) and composition (Figure S2, Supporting Information). In such nanocomposites the impurity ions are statistically distributed over the MgO lattice, which opens up an extremely interesting opportunity region to trigger annealing induced phase separation and to generate novel non‐equilibrium structures.

The nanoparticle powders are made up from agglomerates of monocrystalline and phase pure nanoparticles with periclase structure. Powder XRD patterns of samples with different initial indium concentrations show that vacuum annealing up to T=1173 K increases the crystallite domain size (Figure 1c and d) and leads to particle coarsening, as revelaed by the particle size distribution plots of Figure 1b. The thermal stability of In−Mg−O nanoparticles with indium concentrations as low as c_In_≤2.0 at% are comparable to those of MgO. There is, however, a pronounced In‐induced coarsening effect for samples of higher indium concentrations (i. e. c_In_=10 at%). The powder XRD pattern of In−Mg−O nanoparticles with c_In_≤2.0 at% reveals exclusively periclase‐specific diffraction features. For samples with c_In_=10 at%, annealing of powder samples to T=1073 K and temperatures above leads to additional XRD reflections that originate from In_2_O_3_ in the cubic bixbyite‐structure, whereas there is no evidence for a crystalline MgIn_2_O_4_ phase. Apparently, the impurity segregation and the emergence of a new In_2_O_3_ phase occurs in the range between 873 K≤T≤1173 K and is observed for powder samples with an indium concentration of c_In_=10 at%, where the amount of segregated In_2_O_3_ is sufficient to be detected by XRD.

**Figure 2:**
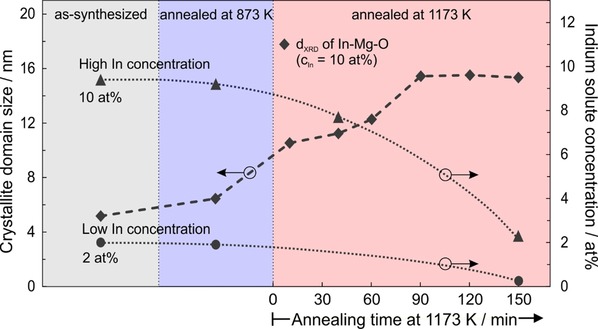
Annealing induced grain growth and In‐depletion of In−Mg−O nanoparticles: (♦) crystallite domain size evolution of In−Mg−O samples (c_In_=10 at%) that were annealed at T=873 K (blue panel) and T=1173 K (red panel) for variable times. The indium concentrations of the as‐synthesized In−Mg−O samples were ▴: c_In_=10 at% and •: c_In_=2.0 at%. At 1173 K there is progressive indium depletion with annealing time (0≤t≤150′).

In addition to and independent from elemental analysis with TEM, energy dispersive X‐ray (EDX) spectroscopy in conjunction with SEM revealed indium concentration changes of In−Mg−O nanoparticle powders after different processing steps. The two independent assessments of the elemental composition consistently show for a processing temperature of T=1173 K an indium depletion effect with time (dotted lines in Figure 2). This is attributed to indium segregation into the particle surface followed by the elimination of a volatile In_2_O_3_ phase via the gas phase. As an evidence for evaporation we observed indium rich deposits in the cold region of the fused quartz cells in which the powder annealing steps were carried out. Indium segregation does also broaden the composition distribution function throughout the sample, as determined with EDX/ SEM with 20 different spots per sample analyzed at the minimum (Figure S2, Supporting Information). After extended annealing at 1173 K the In−Mg−O powders get devoid of indium and the composition distribution function narrows and shifts to a residual indium concentration of a few percent.

The characteristic cubic habit of MgO nanoparticles in anhydrous environments is due to the low surface energies of low index surfaces of alkaline‐earth metal oxides. The surface energy for a bare and dehydroxylated MgO(100) surface has been calculated to be 0.93 J/m^2^, whereas the corresponding values for MgO(110) and MgO(111) with 2.25 and 2.21 J/m^2^, respectively, are substantially higher.^13^,^17^ (The presence of water, however, changes the relative ordering of the surfaces, since the adsorption energies of water to the high index planes are much stronger.)^13^,^18^ Hence, after gas phase synthesis and/ or thermal processing in a dry environment undoped and dehydroxylated MgO particles adopt a cubic shape. Application of processing cycles which alternate between continuous pumping with a base pressure below 10^−5^ mbar and thermal treatment in pure oxygen guarantee such experimental conditions (Figure S3, Supporting Information). In comparison to corresponding materials, which were grown in liquids, mass transfer and sintering is strongly suppressed.^8^,^13^

**Figure 3:**
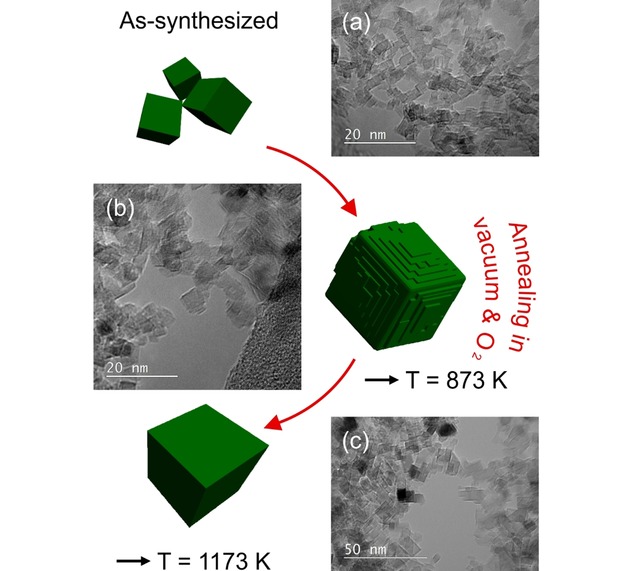
In−Mg−O nanoparticle shape evolution during annealing: TEM images of (a) as‐synthesized In−Mg−O nanoparticles (c_In_=2.0 at%) and nanoparticles annealed at (b) T=873 K and (c) T=1173 K. As‐synthesized nanoparticles where the In^3+^ ions are statistically distributed over the bulk and surface adopt a well‐defined cubic shape. Such type of particle habit is no longer observed on samples after annealing to 873 K, i. e. at a temperature at which substantial ion diffusion has set in. Annealing induced indium depletion in the MgO nanoparticles at 1173 K restores the cubic habit characteristic of pure MgO nanoparticles (c).

Anhydrous In−Mg−O nanoparticle powders with initial indium concentrations in the range between 1 and 2 at% exhibit particle shapes that can be approximated as cubes with sharp edge and corner features as well as flat {100} particle faces (Figure 3a). The TEM image in Figure 3a reveals an additional interesting effect, namely that many of these individual nanocrystals are aligned with respect to each other. This type of orientation relationship arises from electrostatic interaction forces that are active between these highly ionic nanocrystals.

During gas phase synthesis indium ions were trapped and statistically distributed inside the volume of the as‐synthesized MgO nanocrystals. Upon annealing, size and charge mismatch induces their migration into the nanocrystal surfaces where they form a volatile indium oxide phase that evaporates and leads to the depletion of indium in the overall particle powder (dotted lines in Figure 2). In the course of this particle re‐organization process, the particles coarsen and In^3+^ migration into the near surface region exerts an impact on the particles’ surface energies and surface structures. Less regular features such as kinks, step edges and protrusions were observed at many spots of the annealed samples inspected with TEM (Figure 3b). These surface features seem to be favored in the transient transformation state of the individual and metastable nanoparticles of less pronounced cubic habit.

Treatment at higher temperatures promotes further In^3+^ segregation into the particle surfaces and the formation of i) indium deposits in the interparticle regions (see below) and ii) a crystalline In_2_O_3_ phase that evaporates with continued annealing time. As a result, MgO particles that become depleted in indium reorganize via ongoing ion diffusion and re‐adopt their original cubic particle shape.

**Figure 4:**
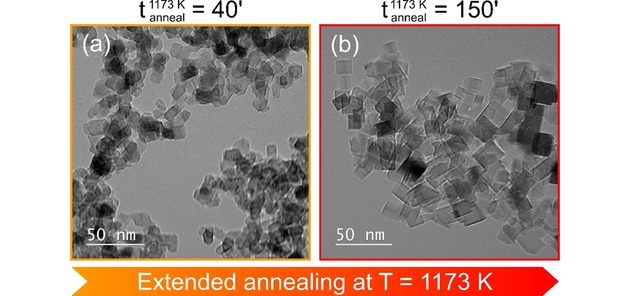
Example for the indium loss induced MgO morphology restoration: TEM images of In−Mg−O nanoparticles (a) after 40 minutes and (b) after 150 minutes of vacuum annealing at 1173 K. The here investigated powder sample had an initial indium concentration of c_In_=10 at%.

A second example for this trend of particle reorganization is related to the morphology evolution observed for In−Mg−O nanoparticles after different times of annealing at 1173 K and in water‐free environment, e. g. by comparing TEM data of powders after annealing for 40 minutes (Figure 4a) and 150 minutes (Figure 4b).

Residual In^3+^ ions in the MgO particle lattice apparently influence the hierarchy of surface energies for the different MgO related faces. As a result, less regular surface features remain on the surface and the particles adopt a roundish shape without sharp edge and corner features. A similar situation was previously observed for Fe^3+^ doped MgO nanoparticles^8^,^16^ where Fe^3+^ ions in the near‐surface region of the nanocrystals promote the formation of terraces, protrusions and step edges of multiple heights. Regular {100} faces being energetically favored in pure MgO nanoparticle systems are significantly less abundant in these doped systems. Extended vacuum annealing leads to further indium removal and promotes a situation where the {100}, {010}, and {001} faces of the particles are again energetically most favored and, hence, represent the most abundant particle surface planes (Figure 4b). Hence, the cubic morphology specific to pure MgO nanoparticles has been re‐established due to vacuum annealing induced indium elimination.

**Figure 5:**
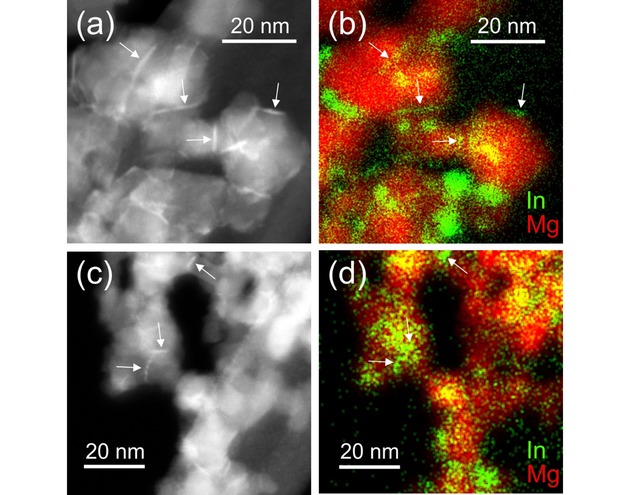
indium segregation and accumulation in intergranular regions between the MgO based particles. HAADF/STEM images (a and c) and corresponding EDX intensity maps (b and d) show that the layers of higher z‐contrast in the intergranular regions (indicated by arrows) appear brighter and are rich in indium (red: Mg, green: In).

Segregation of indium in In−Mg−O samples with an initial solute concentration of c_In_=10 at% were further investigated with STEM and EDX mapping. HAADF/STEM images and the corresponding EDX intensity maps of samples after 40 minutes annealing at 1173 K (see Figure 4a) are shown in Figure 5. White arrows in the HAADF images (5a and c) highlight bright, linear regions, which are attributed to higher z‐contrast between single particles. The higher z‐contrast is related to indium as the corresponding EDX intensity maps (5b and d) clearly prove indium accumulation in these regions. This suggests that In_2_O_3_ is wetting MgO solid‐solid interfaces after annealing. The EDX images also show that in addition to indium in the intergranular regions, there are also clusters of enhanced indium concentration present in annealed samples. This observation gains further support from TEM data obtained on samples after extended annealing, where indium depletion and enforced clustering occurs (see Supporting Information, Figure S4). This means that indium diffusion into intergranular regions represents an intermediate stage of the overall process of indium segregation. With an optimized annealing cycle, systematic decoration of solid‐solid interfaces by indium segregates is achievable for these In−Mg−O particle systems.

**Figure 6:**
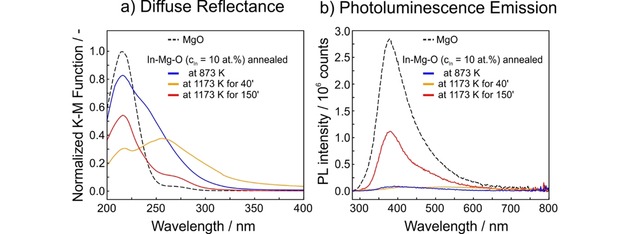
Room temperature optical spectroscopy data of vacuum annealed MgO (at T=1173 K) and three In−Mg−O nanoparticle powder samples (c_In_=10 at%) after annealing treatment as indicated. (a) UV diffuse reflectance spectra recorded in oxygen (p(O_2_)=10 mbar) and (b) photoluminescence emission spectra excited at λ=270 nm acquired in high vacuum. At 1173 K the annealing times were t^1173 K^=40 minutes (orange curve) and t^1173 K^=150 minutes (red curve).

MgO nanoparticles represent a powerful model system for spectroscopic surface and interface studies on particulate metal oxide materials. Optical spectroscopies (UV‐vis diffuse reflectance and photoluminescence emission) can be used to probe the relative abundance of MgO specific surface features, such as corners and edges,^14^,^19^ particle interfaces^20^ and adsorbates.^21^ Previous studies have also shown that the admixture of impurities to the nanoparticle surfaces, e. g. with Ca^2+[22]^ or Zn^2+^ ions^23^ can either modify or quench characteristic photoluminescence emission that originates from the photoexcitation of MgO nanocube edges. In fact, the reorganization of In^3+^ loaded MgO nanoparticles (c_In_=10 at%) can also be monitored via the UV diffuse reflectance spectra, which show a clear dependence on the time and temperature of annealing (Figure 6). A maximum in the Kubelka‐Munk function at λ=220 nm, which can be observed in all spectra, is consistent with the optical absorption of four‐fold coordinated oxygen anions in MgO edges. As a result of annealing at 1173 K with an annealing time interval of t=40′ and subsequent cooling to room temperature, a broad absorption feature between 240 and 320 nm forms and vanishes after extended annealing, i. e. for time intervals of t^1173 K^≥60 minutes and more.

The changes in the sample reflectance at λ>230 nm show that In^3+^ ions as part of the lattice and/or in the form of indium oxide segregates at the particle surface gives rise to new electronic excitations of so far unspecified origin. As the more sensitive technique, photoluminescence spectroscopy was used to probe characteristic emission features originating from the photoexcitation of three‐fold coordinated MgO nanocube corners at λ=270 nm. Consistent with previous studies,^14^ a PL emission band at λ=380 nm was measured for undoped MgO nanocube powders (dashed curve, Figure 6b) as well as for those with admixed indium after extended annealing at T=1173 K (red curve, Figure 6b). The absence of the respective band in the spectra of the In−Mg−O nanoparticle samples with t^1173 K^≤40′ (Figure 6b) indicates that indium either directly at or close to the low‐coordinated sites (MgO‐specific corners and edges) affects the surface electronic structure and effectively quenches the MgO specific photoluminescence.

## Conclusions

Nanoscale grain boundary design is an emerging field in materials science and requires atomic scale insights into electronic and ionic processes that occur within the intergranular regions of nanocrystalline ceramics.^24^,^25^ The results of this study represent an important step towards the knowledge based transformation of vapor phase grown MgO nanocubes into a consolidated network of nanograins with designed particle interfaces and grain boundaries. The low solubility of In^3+^ ions in the bulk of the MgO particles, which arises from size mismatch and its trivalent valence state, drives them into the particle surfaces and interfaces where they wet the faces of the MgO based nanocrystals (Figure 5). In a subsequent step they become gradually depleted upon extended annealing at T=1173 K (Figure 2). We identified a window of process parameters within which impurity segregation can be achieved without indium loss and also obtained first evidences about the wetting behavior of In_2_O_3_ on MgO nanoparticles surfaces.

This exploratory study is about impurity exsolution in oxide nanocrystals, where the impurity serves as a precursor for a segregated semiconducting component, i. e. In_2_O_3_ at the surface of an insulating MgO core component. The approach, trapping of impurities in nanocrystal lattices via gas phase synthesis^8^ in conjunction with control over their subsequent segregation behavior opens the door for new processing concepts towards the surface functionalization of nanoparticle powders. Respective materials can range from nanostructured materials with optically active surfaces^22^ or for the production of nanocrystalline ceramics with engineered grain boundary properties.^25^,^26^

## **Experimental Section**

In−Mg−O nanoparticles were produced in a modified metalorganic chemical vapor synthesis (MOCVS) approach (Figure S1, Supporting Information), which provides high control over concentration and homogeneous distribution of indium in In−Mg−O nanoparticle powders.^8^,^16^ In this approach, an indium organic vapor decomposes in the combustion flame related to the Mg metal vapor oxidation reaction with oxygen inside a hot wall reactor system and under reduced pressures. The two‐zone reactor system (Figure S1, Supporting Information) consists of two quartz glass tubes, which are mounted concentrically inside a heating coil (first heating zone with an operation temperature T1) followed by a ceramic tube furnace (second heating zone with an operation temperature T2). In the first heating zone, a ceramic crucible with a metalorganic In(acac)_3_ (≥99.99%, Sigma Aldrich, 0.2 or 0.75 g) precursor is placed inside the inner glass tube and heated to T1=383 K or 393 K, respectively, in order to sublimate the indium precursor and to adjust its evaporation rate. An argon gas flow (Ar 5.0, volumetric flow rate Q_Ar_=1200 sccm) is guided through the inner tube to transport the metalorganic vapor to the second heating zone where the furnace guarantees a constant temperature T2=913 K. At this position and inside the inner tube a ceramic crucible with Mg metal turnings (99.98%, Alfa Aesar, 1.0 g) leads to sublimation and the generation of metal vapor that becomes mixed with the gaseous indium precursor. The vapor mixture is then transported by the argon gas flow to the end of the inner glass tube, where the vapor mixture gets in contact with molecular oxygen (O_2_ 5.0, Q_O2_=1200 sccm) from the outer tube. indium precursor decomposition inside the combustion flame of Mg vapor leads to subsequent nanoparticle formation. The resulting particles are collected as a powder downstream in a stainless steel net. Operation temperatures (T1 and T2) and pressure (p=70±1 mbar) are kept constant during the entire process of particle collection.

X‐ray diffraction (XRD) measurements were performed on a Bruker AXS D8 Advance diffractometer using Cu Kα radiation (λ=154 pm). Crystalline domain sizes were determined from powder diffraction data using the Scherrer equation.

Transmission electron microscopy (TEM) data were acquired using a TECNAI F20 field emission transmission electron microscope operating at 200 kV equipped with an EDX (energy dispersive X‐ray emission) detector for local composition analysis. Images were recorded using a Gatan Orius CCD camera. TEM grids were prepared by dipping a lacey carbon grid into the powder in order to investigate structural features and composition of material sticking to the grid.

Scanning transmission electron microscopy (STEM) images, such as secondary and back scattered electron or high‐angle annular dark‐field (HAADF) images, showing z‐contrast were acquired using a cold field emission JEOL F200 STEM/TEM operated at 200 kV equipped with a large windowless JEOL Centurio EDX detector (100 mm^2^, 0.97 srad, energy resolution <133 eV). EDX maps were acquired with a typical beam current of 0.1 nA and a beam diameter of 0.16 nm during 10–30 minutes. The maps were obtained by signal integration of counts over Mg Kα transition line for Mg (integration: 1.16–1.34 keV) and In Lα line for In (integration: 3.15–3.41 keV).

After different processing steps (Figure S2, Supporting Information) we determined the indium concentration of the powder samples using a Zeiss Gemini Ultra 55 scanning electron microscope (SEM) operating at 20 kV equipped with an Oxford EDX‐Silicon drift detector (50 mm^2^). EDX spectra were obtained here by signal integration of counts over Mg Kα transition line for Mg (integration: 1.12–1.37 keV) and In Lα line for In (integration: 2.78–4.28 keV). The elemental compositions of 20 different sample spots at the minimum were determined to evaluate the compositional homogeneity of the nanoparticle powder.

UV‐vis diffuse reflectance (UV‐vis DR) spectra of In−Mg−O powders were recorded with a Perkin Elmer Lambda 750 spectrometer equipped with an integrating sphere (d=60 mm, Spectralon). A high vacuum tight fused silica cell, which allows for optical measurements at pressures in the range between 5 ⋅ 10^−6^<p<10^3^ mbar and under defined gas atmospheres was used for spectroscopic measurements. UV‐vis DR spectra were recorded in the presence of 10 mbar O_2_ in order to quench surface exciton related luminescence from particle surfaces^14^ and, hence, to avoid luminescence related artefacts in the absorption spectra of the samples.

Photoluminescence (PL) emission spectra were recorded on a FLS980 PL spectrometer from Edinburgh Instruments. A measuring assembly with a front‐facing sample holder suitable for powder samples was used and measurements were performed using an excitation wavelength of 270 nm, a slit width of 2 nm and an integer value of 2 nm.

## Conflict of interest

The authors declare no conflict of interest.

## Supporting information

As a service to our authors and readers, this journal provides supporting information supplied by the authors. Such materials are peer reviewed and may be re‐organized for online delivery, but are not copy‐edited or typeset. Technical support issues arising from supporting information (other than missing files) should be addressed to the authors.

Supplementary
