# Supplementary material for: Impurity Segregation and Nanoparticle Reorganization of Indium Doped MgO Cubes
Source: ChemNanoMat. 2019 Apr 3;5(5):634–41. doi: 10.1002/cnma.201900077 (PMC6563704; doi:10.1002/cnma.201900077)
Supplement: Supplementary file 1 — Supplementary [file CNMA-5-634-s001.pdf]

# Supporting Information

© Copyright Wiley-VCH Verlag GmbH & Co. KGaA, 69451 Weinheim, 2019

## Impurity Segregation and Nanoparticle Reorganization of Indium Doped MgO Cubes

Matthias Niedermaier, Chatpawee Taniteerawong, Thomas Schwab, Gregor Zickler, Johannes Bernardi, and Oliver Diwald*© 2019 The Authors. Published by Wiley-VCH Verlag GmbH & Co. KGaA.
This is an open access article under the terms of the Creative Commons Attribution License, which permits use, distribution and reproduction in any medium, provided the original work is properly cited.

# Impurity Segregation and Nanoparticle Reorganization of Indium doped MgO Cubes

Matthias Niedermaier[1], Chatpawee Taniteerawong[1], Thomas Schwab[1], Gregor Zickler[1], Johannes Bernardi[2], and Oliver Diwald[1]*

[1] Department of Chemistry and Physics of Materials, University of Salzburg, Jakob-Haringer-Strasse 2a, 5020 Salzburg, Austria.
[2] University Service Centre for Transmission Electron Microscopy, Technische Universität Wien, 1040 Vienna, Austria.

E-mail: oliver.diwald@sbg.ac.at

## Electronic Supplementary Information

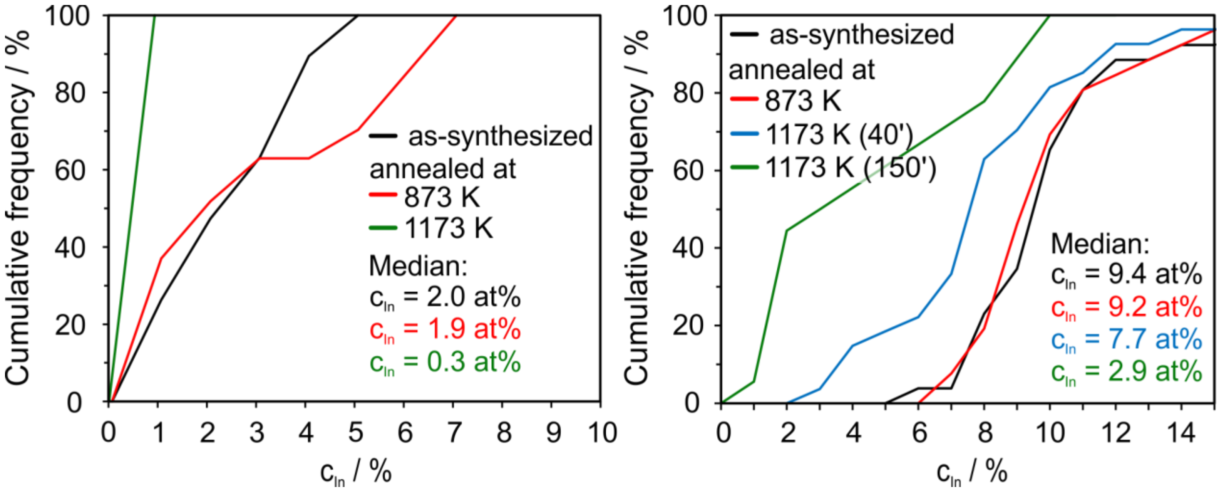


**Figure S1**: Composition distribution functions obtained from EDX spectroscopy on In-Mg-O samples either as-synthesized or after thermal treatment at T = 873 or 1173 K (t = 40' or 150'). EDX spectroscopy was performed on 20 spots of the different samples at the minimum to evaluate homogeneity of the samples.

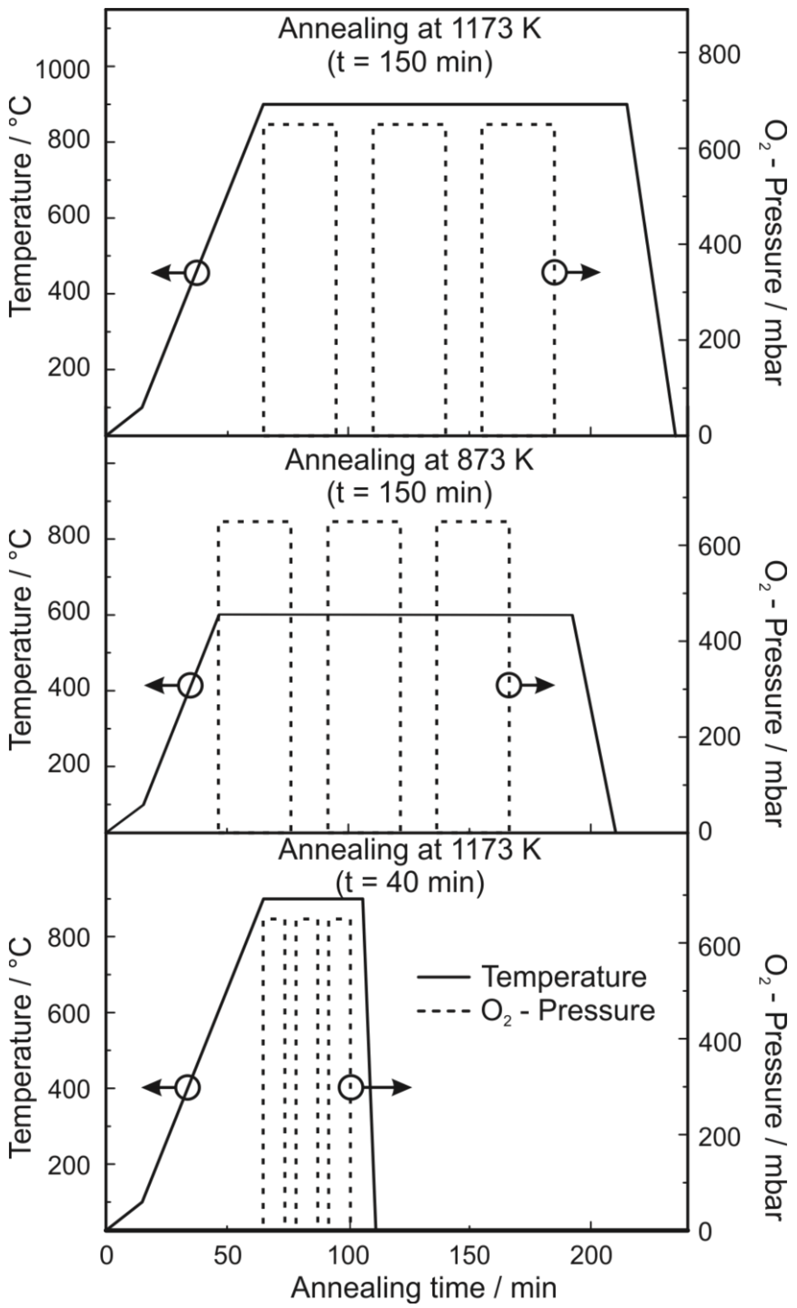


**Figure S2**: Annealing cycles with different final temperatures (T = 873 K or 1173 K) and variable dwell times at T (t = 40' or 150'), each with a high vacuum step to the final temperature and three subsequent annealing cycles under $O_2$ atmosphere ($pO_2$ = 650 mbar).

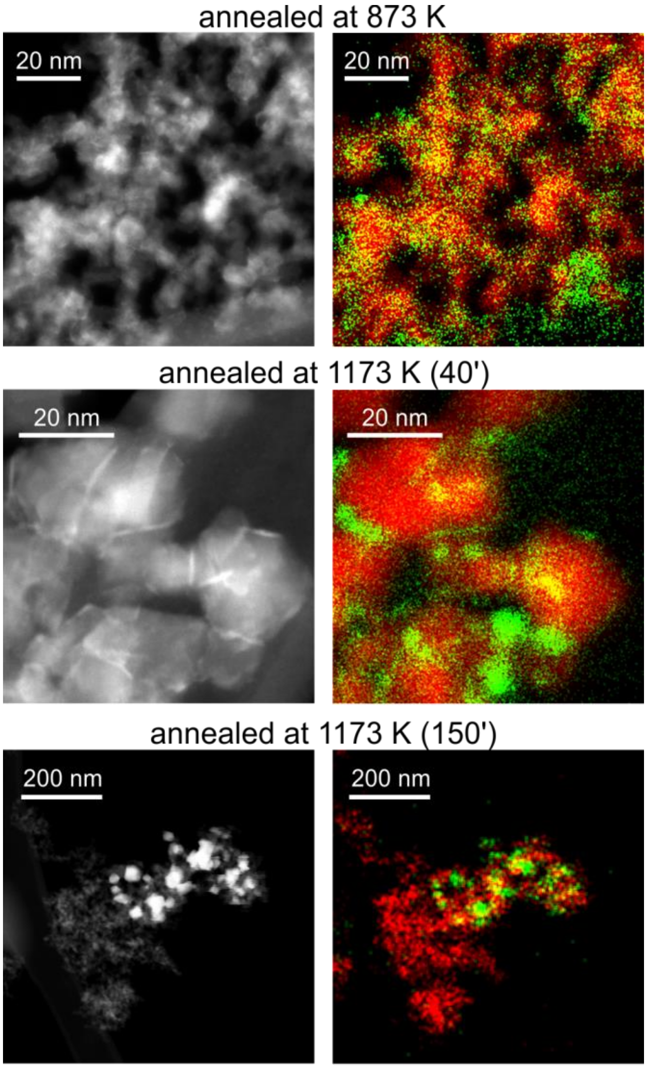


Figure S3: Indium distribution over In-Mg-O nanoparticles after different thermal treatment at T = 873 or 1173 K (t = 40’ or 150’). HAADF/STEM images (left column) and corresponding EDX intensity maps (right column; red: Mg, green: In) showing Indium segregation during annealing at T = 1173 K.
